# Reoperation for chronological complete dislodgement of the bioprosthetic aortic valve into the left ventricle due to Takayasu arteritis

**DOI:** 10.1186/s44215-024-00182-2

**Published:** 2024-12-31

**Authors:** Shogo Matsunaga, Hiromichi Sonoda, Tomoki Ushijima, Meikun Kan-o, Satoshi Kimura, Akira Shiose

**Affiliations:** Department of Cardiovascular Surgery, Kyushu University Hospital, 3-1-1 Maidashi, Higashi-Ku, Fukuoka, 812-8582 Japan

**Keywords:** Aortic valve replacement, Dislodgement of the bioprosthetic valve, Takayasu arteritis, Paravalvular leakage, Structural valve deterioration

## Abstract

**Background:**

Takayasu arteritis is a large-vessel vasculitis, in addition to giant cell arteritis. Various post-operative complications associated with the cardiac macrovasculature have been reported. Detachment of the prosthetic valve, pseudoaneurysm formation, and dilatation of the aortic root are well-known post-operative complications associated with vasculitis syndromes, including Takayasu arteritis. Here, we report a rare complication involving aortic bioprosthetic valve dislodgement in the left ventricular outflow tract due to Takayasu arteritis.

**Case presentation:**

A 76-year-old female underwent aortic valve replacement with a 21-mm Carpentier–Edwards Perimount valve for severe aortic regurgitation and a coronary artery bypass graft from the left internal thoracic artery to the left anterior descending artery for ischemic heart disease. Fourteen years after the initial surgery, echocardiography revealed severe aortic valve sclerosis due to structural valve deterioration of the bioprosthesis. Upon scrutiny, the bioprosthetic aortic valve was found to have dislodged into the left ventricular outflow tract. We performed re-implantation of the bioprosthetic aortic valve and replacement of the ascending aorta.

**Conclusions:**

Although dislodgement of the bioprosthetic aortic valve is an extremely rare complication associated with Takayasu arteritis, the possibility that it could occur should be considered when treating the post-operative patients.

## Background

Takayasu arteritis (TA) is an autoimmune disease classified as a large-vessel vasculitis, in addition to giant cell arteritis. Various cardiovascular complications result from impaired blood flow due to the narrowing, occlusion, and dilatation of blood vessels, typically affecting the aorta and its major branches, the aortic valve, coronary arteries, and pulmonary arteries [1]. Aortic regurgitation due to TA occasionally requires surgical intervention, such as aortic valve replacement (AVR) and composite graft root replacement. However, surgical interventions for vasculitis syndrome present many difficulties, not only because of the necessity to manipulate fragile and inflamed tissue, but also because of post-operative complications, such as detachment of the prosthetic valve with paravalvular leakage, pseudoaneurysm formation at the suture line, and annuloaortic ectasia, which are often encountered during the early or late post-operative periods. Herein, we report the rare complication of chronological dislodgement of a bioprosthetic aortic valve completely into the left ventricle due to TA.

## Case presentation

**Fig. 1:**
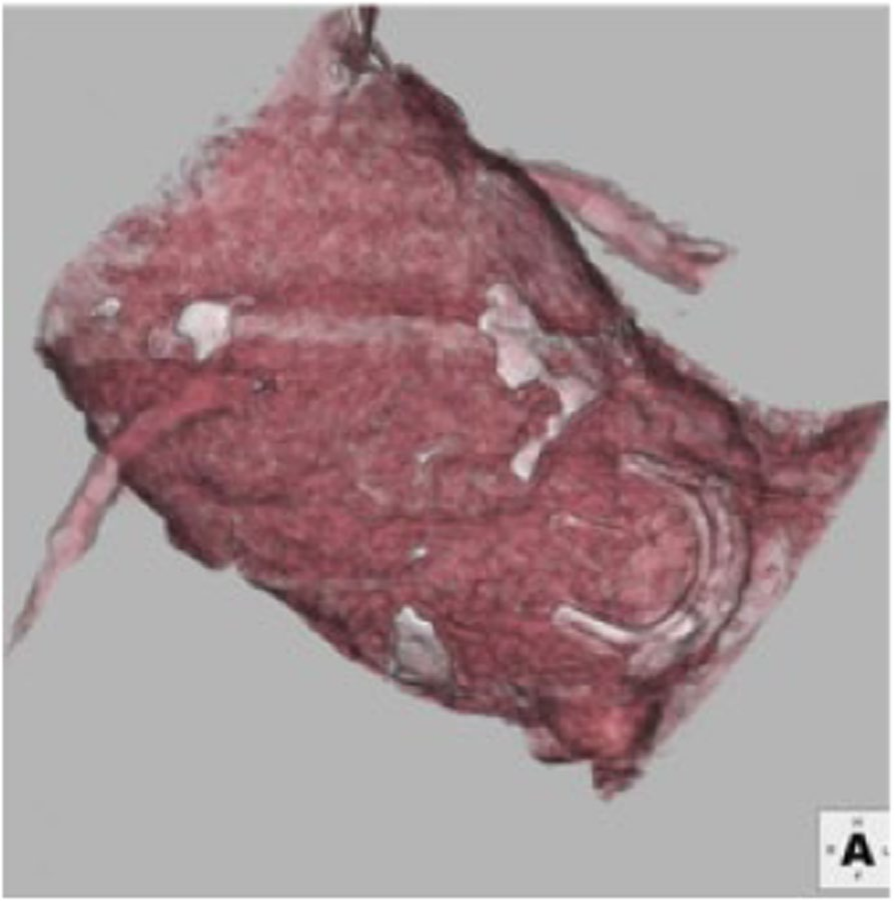
The 3D computed tomography image showed dislodgement of the bioprosthesis entirely approximatley 20 mm beneath the aortic annulus into the left ventricle outflow tract

A 76-year-old female underwent AVR with a 21-mm Carpentier–Edwards Perimount valve (Edwards Lifesciences, Irvine, CA, USA) and a coronary artery bypass graft for aortic regurgitation with ischemic heart disease at another hospital. At the initial operation, TA was suspected based on the thick and distorted aorta; however, pathological assessment did not lead to a definitive diagnosis. She received neither steroids nor immunosuppressive drugs during the post-operative period. She underwent ablation for paroxysmal atrial fibrillation the following year, and permanent pacemaker implantation for complete atrioventricular block was performed 2 years after the initial operation. During therapy for right heart failure associated with pulmonary hypertension, she presented with congestive heart failure due to aortic sclerosis caused by structural valve deterioration (SVD) 14 years after the initial operation. Transthoracic echocardiography revealed thickened and immobile valve leaflets in the bioprosthesis. Elevations in the mean transaortic gradient (40 mmHg) and peak transaortic velocity (4.1 m/s) were compatible with severe aortic sclerosis due to SVD. Computed tomography revealed a distorted and dilated ascending aorta with a maximum diameter of 42 mm. In addition, malpositioning of the implanted bioprosthesis, approximately 20 mm beneath the original aortic annulus into the left ventricle outflow tract, was discovered (Fig. 1). Surgical reintervention was indicated. During the operative period, she did not complain of fever, and preoperative blood examination showed no elevation in serum C-reactive protein levels, suggesting inactive inflammation. Fluorodeoxyglucose-positron emission tomography also showed no abnormal accumulation in the aorta, and the aortic annulus appeared negative in the active phase of TA. Because she had complicated Child–Pugh class B cirrhosis with ascites and potential pulmonary hypertension, treated with home oxygen therapy, the estimated mortality was calculated to be 48.73% and 90.6% using the STS PROM score and EuroECORE II, respectively.

**Fig. 2:**
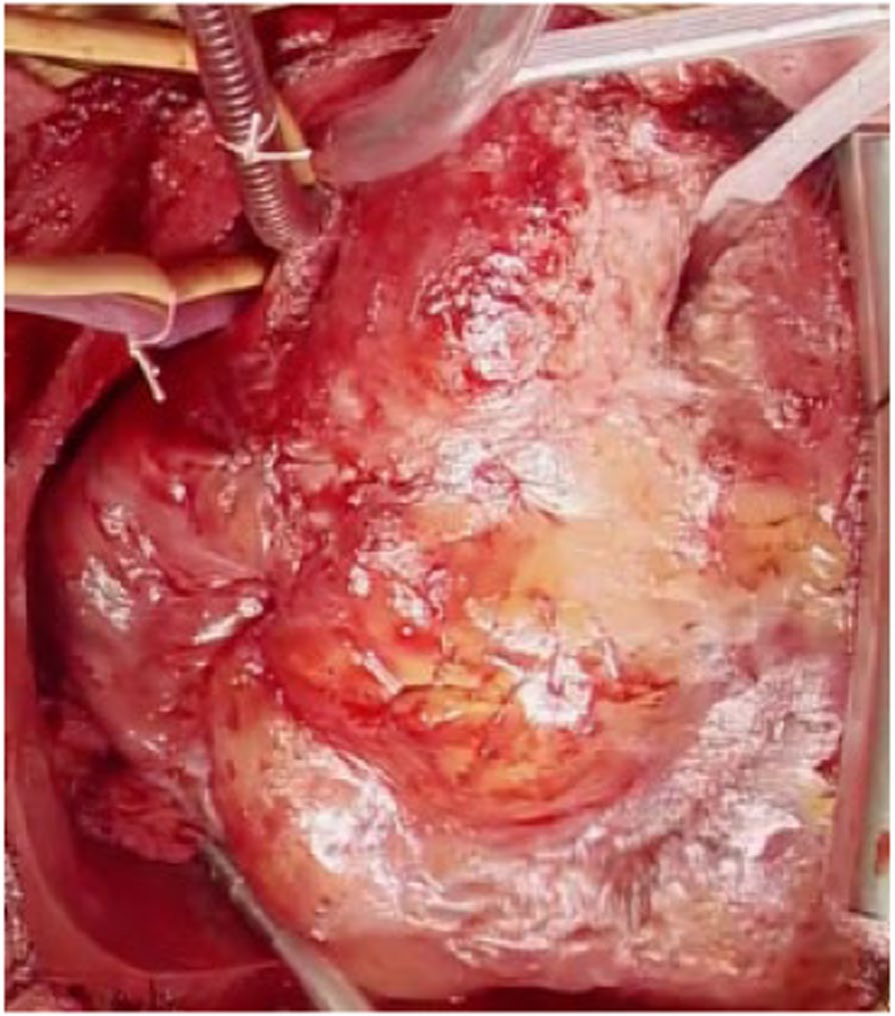
Operative findings showed that the proximal ascending aorta and aortic root were severely adherent and showed distorted deformity

**Fig. 3:**
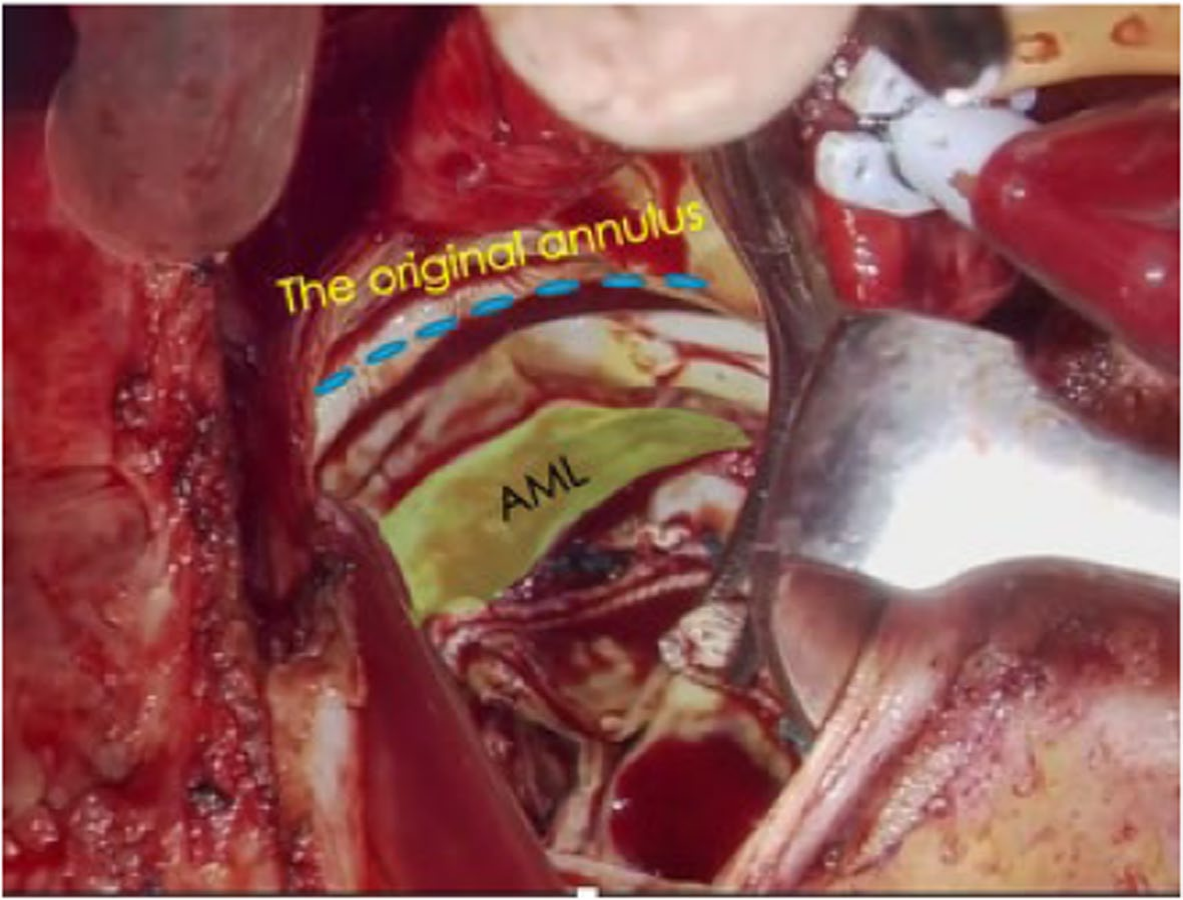
Operative findings showed that the bioprosthetic valve had been dislodged from the annulus into the left ventricular outflow tract and was firmly fixed on the aortic–mitral continuity. A faint right non coronary cusp commissure had remained as the annulus tissue

Redo AVR and replacement of the ascending aorta were performed via a median resternotomy. The proximal ascending aorta and aortic root were severely adherent and distorted (Fig. 2), possibly due to post-inflammatory reactions related to TA. After aortic clamping and cardioplegic arrest, inspection of the inside of the aortic root showed that the bioprosthesis, which had been implanted in the supra-annular position, was completely dislodged from the annulus into the left ventricular outflow tract and fixed tightly (Fig. 3). Massive adhesions around the right coronary artery with calcification led us to decide not to perform composite graft root replacement. Despite the majority of the annular tissue being missing, the partial annulus around the right/non-commissure remained healthy, enabling AVR with replacement of the ascending aorta. The degenerated bioprosthesis with residual stitches and some previously used pledgets were carefully explanted, and a 27-mm Inspiris Resilia aortic bioprosthetic valve (Edwards Lifescience, Irvine, California, USA) was implanted with everting horizontal mattress sutures to the remaining annulus tissue and to the area where annulus tissue was missing by suturing directly to all layers of the aortic wall deeply. The ascending aorta was replaced with a straight J-Graft 30 mm (Japan Lifeline Co., Ltd, Tokyo, Japan).

During the post-operative course, transthoracic echocardiography revealed normal valve function without paravalvular leakage or rocking motion of the bioprosthesis. This patient required tracheotomy because of post-operative interstitial pneumonia; however, medication and intense respiratory rehabilitation enabled the removal of the tracheal tube and the transfer to another hospital for rehabilitation. The patient currently lives independently in the facility.

## Discussion and conclusions

Bioprostheses are prone to SVD, which results in limited long-term durability. Medication strategies for mild-to-moderate aortic sclerosis or paravalvular leakage caused by SVD are acceptable. However, symptomatic heart failure due to aortic sclerosis or severe hemolytic anemia with paravalvular leakage necessitates surgical intervention. There were some surgical strategies for this case; transcatheter aortic valve (TAV)-in-surgical aortic valve (SAV) implantation should be considered because of the extremely high operative risk [2]. However, the dislodgement of the bioprosthetic valve from the original annulus was not considered adaptive. The post-operative management of aortic regurgitation associated with TA can be complicated, with the occurrence of prosthetic valve detachment, formation of pseudoaneurysms along the suture line, and progressive dilatation of the aortic root [3, 4]. However, the complications of aortic bioprosthetic valve dislodgement in the left ventricular outflow tract are extremely rare. Composite graft root replacement for this repeat procedure is preferable. Matsuura et al. compared aortic valve replacement alone or composite graft root replacement for aortic regurgitation with some root dilatation due to TA at the initial operation and concluded that the latter was superior in the incidence of valve or graft detachment [5]. However, this case was a redo case with extremely high operative risks and needed to be less invasive. Fortunately, the inflammation due to TA had been in an inactive phase, we decided to performed AVR and replacement of the ascending aorta without adhesiotomy around the annulus and coronary arteries.

**Fig. 4:**
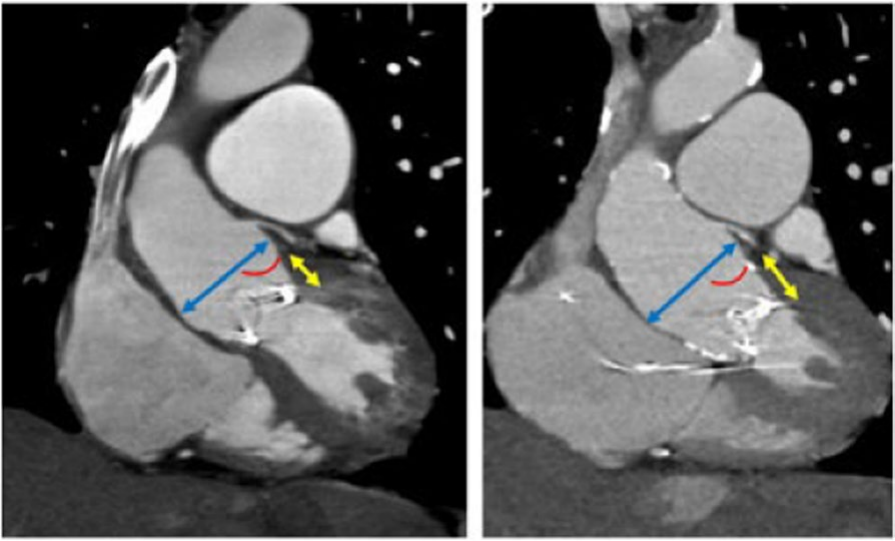
Computed tomography images revealed the malposition of the bioprosthesis, and gradual increase in diameter between the original annulus line and the bioprosthesis. (Rt) 1 year after the initial surgery, (Lt) 13 years after the initial surgery

Malpositioning of a bioprosthetic valve can be diagnosed based on several hypotheses. First, there is the possibility that a bioprosthetic valve was implanted at another location from the annulus during the initial operation. Post-operative transthoracic echocardiography reports of the initial operation indicated normal valve function, and malpositioning of the bioprosthesis was not detected (data not shown). The operative finding of a residual partial right/non-commissure also denied this possibility. Second, it is possible that the tissue between the annulus and sinus of Valsalva gradually elongated. Operative findings also showed that the bioprosthetic valve was firmly fixed on the aortic–mitral continuity, indicating no elongation but dislodgement into the left ventricular outflow tract. In a retrospective assessment, computed tomography images revealed that malpositioning had already occurred 1 year after the initial surgery. Subsequently, the diameter between the original annulus line and bioprosthesis gradually increased (Fig. 4).

**Fig. 5:**
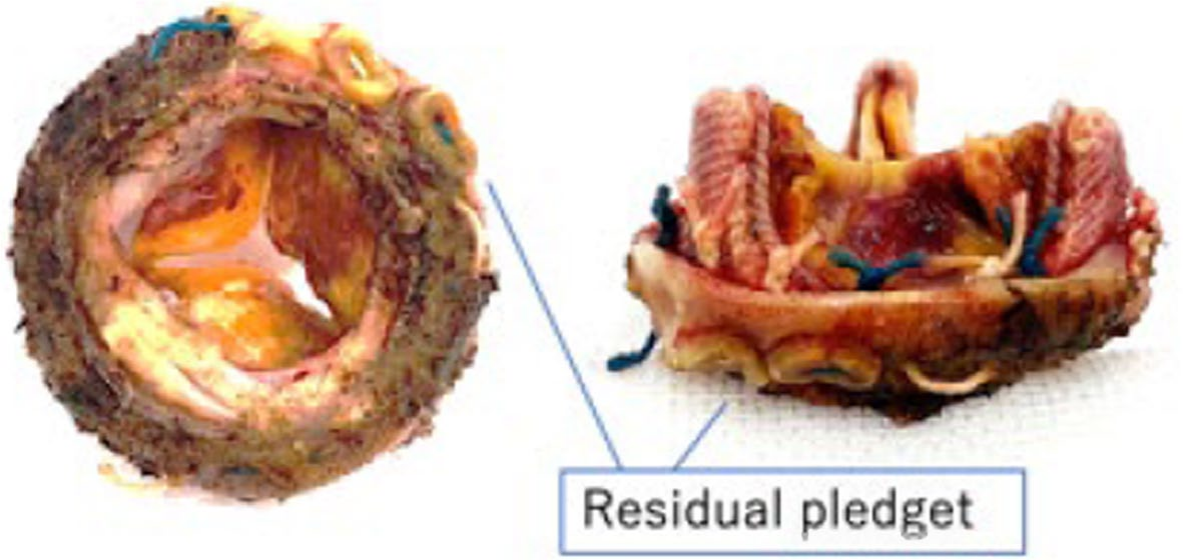
The explanted bioprosthesis with residual stitches and some previously used pledgets

However, the mechanisms underlying chronological dislodgement remain unclear. One possibility is that all stitches from the initial operation were cut entirely, but the carefully explanted bioprosthesis retained residual stitches with some previously used pledgets (Fig. 5). This suggests the possibility that the annulus itself split and migrated with the implanted bioprosthesis due to inflammation, and the fragility of the annulus related to TA enabled this phenomenon. During follow-up, transthoracic echocardiography revealed no paravalvular leakage or rocking of the bioprosthesis. In the case of paravalvular leakage associated with prosthetic valve detachment, diastolic pressure from blood flow is weighted toward either side of the bioprosthesis, leading to a rocking motion. However, in the present case, without paravalvular leakage, diastolic pressure would apply to the entire bioprosthesis and annulus, leading to gradual dislodgement of the bioprosthesis and annulus itself.

We report a rare complication of chronological dislodgement of a bioprosthetic aortic valve into the left ventricle due to fragility associated with TA. Considering the various vascular complications associated with TA, careful post-operative assessment should be performed.

## Data Availability

The data that support the findings of this study are available from the corresponding author, [Akira Shiose, MD, PhD], upon reasonable request.

## Abbreviations

TA: Takayasu arteritis
AVR: Aortic valve replacement
SVD: Structural valve deterioration
